# Improving the approval of jumping techniques on the balance beam focusing on the “switch leap to ring position”

**DOI:** 10.3389/fspor.2026.1798778

**Published:** 2026-05-04

**Authors:** Misato Ishikawa, Yune Yasu, Kazuyuki Koizumi, Toshio Yanagiya, Keiichiro Hata, Hiroyuki Tomita, Olga Kozachenko, Shota Hisanaga, Shogo Nonomura, Zhe Sun, Toshiaki Oda, Hidefumi Waki, Mutsumi Harada

**Affiliations:** 1 Graduate School of Health and Sports Science, Juntendo University, Chiba, Japan; 2 Graduate School of Health Data Science, Juntendo University, Chiba, Japan; 3 Faculty of Health and Sports Science, Juntendo University, Chiba, Japan; 4 Faculty of Physical Education, International Pacific University, Okayama, Japan; 5 Institute of Sports Sciences, International Pacific University, Okayama, Japan; 6 Faculty of Health Care and Nursing, Juntendo University, Chiba, Japan; 7 Faculty of Education, Kobe Shinwa University, Hyogo, Japan

**Keywords:** artistic gymnastics, balance beam, dance elements, elements recognition, judging support system, switch leap to ring position

## Abstract

This study focused on the switch leap to ring position in women's artistic gymnastics (i) to elucidate its technical characteristics and (ii) to exploratorily identify key factors, beyond basic technical criteria, that may contribute to consistent recognition. AI-based judging support system (JSS) decisions were compared with those of certified human judges using 46 borderline trials from four collegiate gymnasts. Results showed that trials recognized by both the JSS and judges (*n* = 5) exhibited larger split angles (186.8 ± 5.9°) and higher rear-toe apexes (7.0 ± 4.4 cm) than unrecognized trials. In addition to these rule-defined variables, the rear knee was consistently maintained above hip level. While the JSS provided a numerical baseline, discrepancies with judges suggested the relevance of additional features such as rear-knee elevation. Qualitative observations suggested that positioning the arms posterior to the head might enhance visual arch continuity, potentially influencing human perception independently of coordinate-based logic. These findings indicate that recognition depends not only on numerical thresholds but also on a visually coherent “gestalt” posture. Accordingly, enhancing the coordination and visibility of key morphological features could be a beneficial factor to increase recognition likelihood.

## 1 Introduction

Artistic gymnastics is a subjectively judged sport in which routines are scored to determine rankings. Under the Code of Points of the International Gymnastics Federation (FIG), the total score is the sum of the difficulty score (D-score), execution score (E-score), and any neutral deductions (e.g., overtime or out-of-bounds) (1). Scoring relies primarily on judges’ visual check: certified judges identify elements and evaluate execution based on their training and experience. In recent years, advancements in apparatus and techniques have substantially increased the difficulty of routines, making it challenging to perceive and judge in real-time the joint angles and body positions specified in the FIG Code of Points, which can compromise scoring accuracy (2). Moreover, results are reported to three decimal places because of panel averaging; thus, even very small differences are likely to influence the final rankings. Accordingly, ensuring the accuracy and consistency of judging is essential at major international competitions. Previous studies have shown that judging can be influenced by observation position and that judges' accuracy varies systematically across disciplines and performance levels (3, 4). Video review using fixed-position cameras (typically limited to the side view) has been introduced to support judging; nevertheless, camera placement and field-of-view constraints can leave parts of the athlete's body unobservable. Consequently, side-view footage alone has inherent limitations for accurate element evaluation.

To address these challenges, Fujitsu Ltd., in collaboration with the FIG and the Japan Gymnastics Association (JGA), developed a judging support system (JSS) based on artificial intelligence (AI) (2). Despite being markerless, the system infers athletes’ full-body three-dimensional joint positions (skeletal coordinates) from synchronized multicamera video and recognizes elements using pretrained models. The system was officially introduced at the 2019 World Artistic Gymnastics Championships in Stuttgart and now covers all apparatus in both men's and women's artistic gymnastics. According to Fujitsu Ltd., the JSS is designed to complement judges’ human assessments and enhance scoring accuracy, consistency, and fairness. Nevertheless, the derived three-dimensional skeletal data have broader potential applications, such as biomechanics-based performance analysis and coaching support.

Importantly, while AI-based technologies are increasingly discussed in sports science (5), previous research on the JSS has been limited to qualitative explorations of stakeholders’ perceptions (6), and scholarly investigations utilizing actual data from the JSS remain limited. Although the relationship between human judgment and objective kinematic data has been demonstrated in other judged sports such as dancesport (7), the potential discrepancies between automated numerical recognition and human judging perception in gymnastics have not yet been fully elucidated.

For elements that are particularly difficult to recognize, quantitatively characterizing and visualizing posture changes can help delineate their technical characteristics and clarify factors related to recognition or nonrecognition. In addition, such analyses may inform performance enhancement and judges’ education. Nevertheless, although the Code of Points and Help Desk clarifications specify recognition requirements for each element, in actual competitions, noncodified factors, such as the positioning of the arms and legs, may influence whether an element is recognized, in addition to codified criteria, such as split angle or an arched position (8). These influences may contribute to inter-judge variability and inconsistencies in the application of decision criteria.

Among elements for which judges’ decisions often diverge, this study focused on the switch leap to ring position on the balance beam. Routines on the balance beam consist of both acrobatic elements (dynamic skills involving somersaults and twists) and dance elements (jumps and turns emphasizing flexibility and artistic expression). As a dance element, the switch leap to ring position is evaluated not only according to codified technical criteria such as split angle and trunk position but also in relation to aesthetic qualities associated with posture and bodily expression.

In women's artistic gymnastics, dance elements are evaluated not only according to codified technical criteria such as split angle and trunk position, but also within an aesthetic context involving posture and bodily expression (15). Empirical research further indicates that larger split angles and more vertically aligned body postures are consistently perceived as more aesthetic across different observer groups (9). Thus, posture plays a central role in both technical recognition and aesthetic evaluation. In particular, aesthetic features like arm positioning might contribute to a perception of movement “completeness,” which could potentially be associated with a judge's recognition decision in borderline cases.

**Figure 1:**
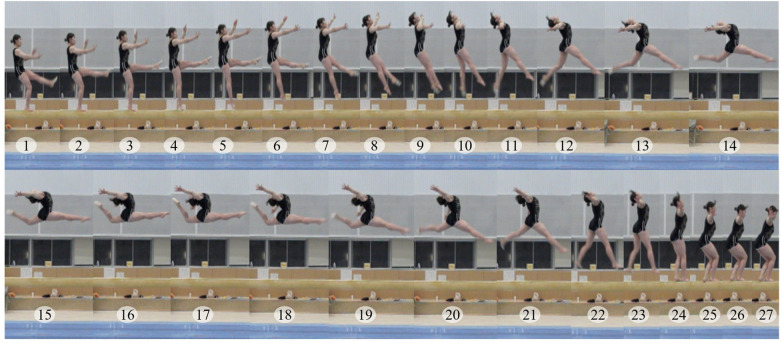
Sequential frames of the switch leap to ring position on the balance beam.

The switch leap to ring position is classified as an E-difficulty element within the dance group and is strategically incorporated into elite-level routines to increase the D-score. However, if the defining ring posture is not recognized, the element is downgraded to a C-difficulty switch leap, potentially affecting the difficulty composition. The element is performed by taking off from one foot, passing through a crossed front-split position, and forming a ring-like configuration in midair by markedly arching the trunk and drawing the rear leg toward the head (Figure 1). Because this defining posture appears only momentarily during the flight phase and involves a dynamically changing combination of trunk hyperextension and rear-leg elevation, judges must visually capture a brief and complex body configuration to determine whether recognition criteria are fulfilled. These structural characteristics make the element particularly recognition-sensitive and therefore an appropriate target for quantitative investigation.

In the present study, We hypothesized that the recognition of the switch leap to ring position is associated not only with the attainment of codified technical criteria but also with the integrated configuration of multiple body components. Additionally, since the JSS relies on numerical thresholds rather than overall impression, we expected it to result in a higher recognition rate than human judges in borderline cases.

Accordingly, this study focused on the switch leap to ring position, an element within the dance group of women’s artistic gymnastics that frequently results in divergent recognition outcomes, (i) to elucidate its technical characteristics and (ii) to exploratorily identify key factors, beyond basic technical criteria, that may contribute to consistent recognition.

## 2 Methods

### 2.1 Data collection

Data were collected from June 12 to 17, 2023. Participants were four female collegiate artistic gymnasts (aged 18.3 ± 0.5 years; height, 150.8 ± 4.8 cm; competitive experience, 13.0 ± 1.2 years), all of whom had experience competing at national-level competitions in Japan and incorporated the switch leap to ring position into their balance-beam routines. They trained six days per week as part of their regular collegiate training programs. Athlete selection was conducted by a coach holding a JGA Class 1 judge certification, based on verified execution of the element in official competitions.

Before participation, participants were fully informed about the study's purpose and significance, procedures, and potential risks, and they provided written and verbal informed consent. The study protocol was approved by the Ethics Review Committee of the Graduate School of Health and Sports Science, Juntendo University (approval no. Jundai-S 2022-139).

**Figure 2:**
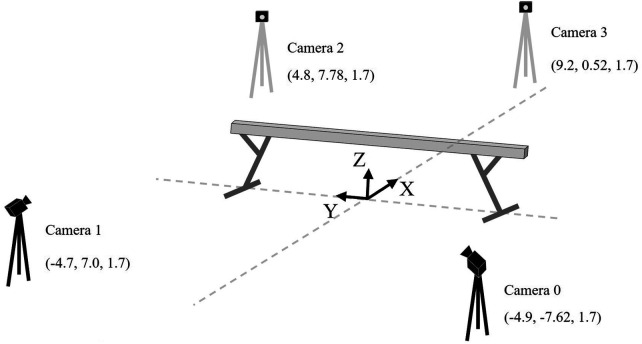
Experimental setup and camera layout. Camera positions are indicated by **(X,Y,Z)** coordinates in m. All cameras were mounted at a consistent height of 1.7 m. Dashed lines represent auxiliary lines indicating the floor-level locations of each camera.

All trials were performed during regular practice sessions after the athletes had completed their usual warm-up routines. During practice, each gymnast performed the switch leap to ring position on the balance beam, yielding a total of 110 trials. No additional task-specific instructions were provided. Performances were recorded with four dedicated JSS cameras (30 Hz; Baumer VCXG-23C, Germany). The camera arrangement followed the standard protocol of Fujitsu Ltd. for balance beam analysis (Figure 2). To ensure temporal consistency, camera synchronization was achieved via the Action Command feature of the Baumer SDK, which issued simultaneous shutter triggers to all cameras over a dedicated, isolated network. Calibration followed the protocol specified by Fujitsu Ltd.Video data were processed using Fujitsu's JSS, which automatically estimated three-dimensional skeletal coordinates and detected the element based on postural changes. The JSS evaluates elements based on FIG technical standards, as described in Fujitsu's official documentation (10).

### 2.2 Evaluation of the element

**Figure 3:**
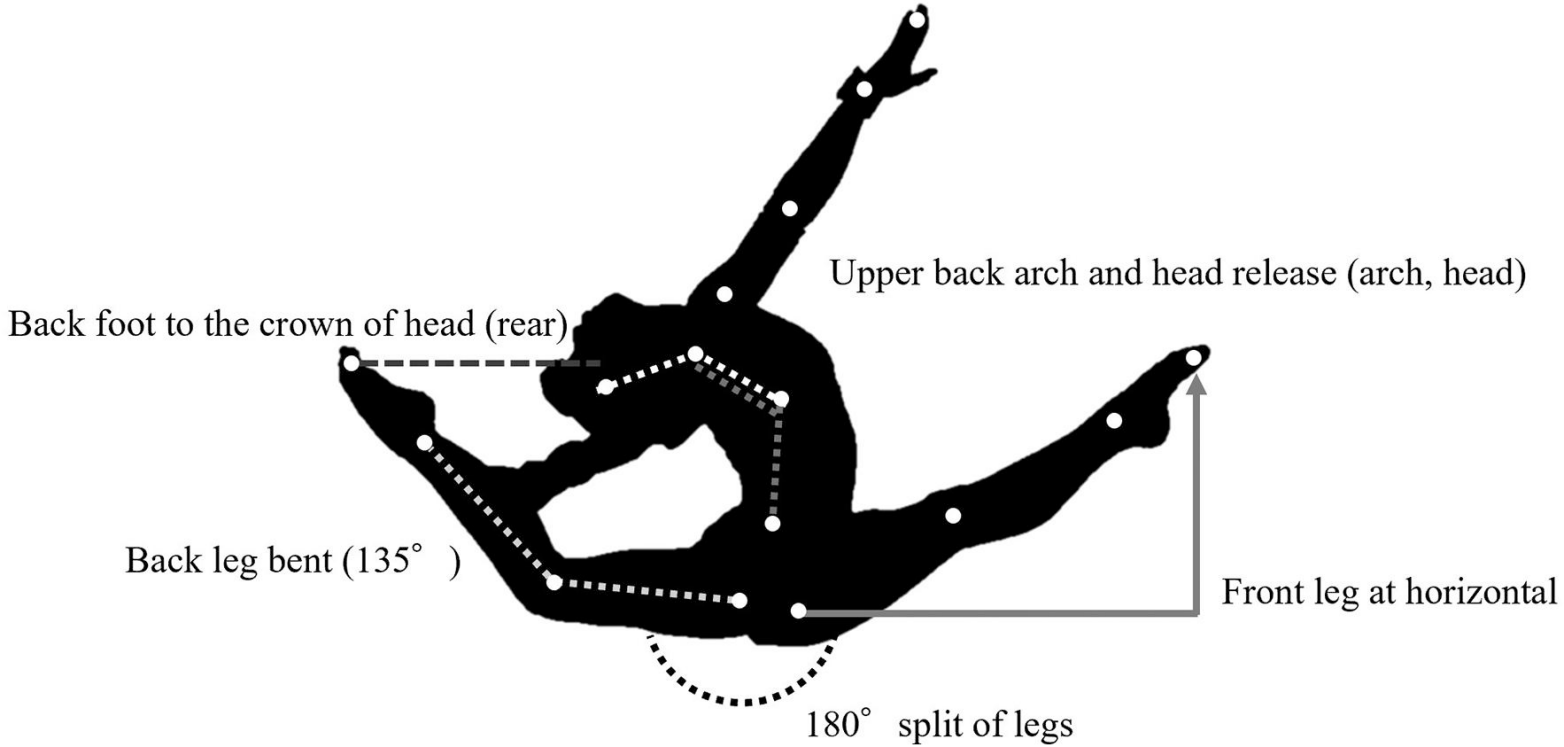
Judging criteria for the switch leap to ring position.

In this study, the recognition criteria for the switch leap to ring position were defined based on the FIG requirements (Figure 3). The primary recognition criteria were as follows:

(1) Split angle ≥170° (split)
(2) A clearly evident arched posture (arch) and head extension (head)
(3) Apex of the rear leg at or above the height of the vertex of the head (rear)
(4) Rear-knee flexion angle (rear angle)
In addition, informed by feedback from judges and athletes, the following supplemental factor was considered:
(5) Position of the rear knee

Although the specific element-recognition logic and decision algorithms implemented in the JSS are proprietary, Fujitsu, in collaboration with FIG, continues to refine AI-based recognition standards. The variables are summarized in Table 1; these correspond to items available on the dedicated JSS terminal.

**Table 1:** Definition of measurement items in the switch leap to ring position.

Measurement items	Definition
Arch angle	Angle obtained by combining the chest and neck extension angles
Rear foot position	Vertical distance from the horizontal line through the vertex to the rear toe
Rear knee angle	Angle of knee flexion of the rear leg
Rear knee position	Vertical distance from the horizontal line through the hip joint to the rear knee
Split angle	Angle between both thighs across the hip joints

As a pre-screening step, one coach who holds a JGA Class 1 judge certification reviewed all 110 trials based solely on the FIG recognition criteria described above and classified them into three categories. Trials that clearly satisfied all primary criteria were classified as (A) recognized, whereas trials that clearly failed to meet one or more primary criteria were classified as (B) not recognized. Trials in which at least one primary criterion was marginally close to the threshold during the coach's pre-screening, making them potentially subject to disagreement among judges, were classified as (C) borderline.

The 46 trials classified as borderline were then presented to other certified judges. The distribution of these 46 borderline trials across athletes was 15 (32.6%), 6 (13.0%), 17 (37.0%), and 8 (17.4%), respectively. The trials were shown in a randomized order and were evaluated under conditions equivalent to official competition judging. Each trial was viewed once only, without the possibility of replay or slow-motion viewing, with judges blinded to the JSS results. The judges responded to the following items:

- Recognition decision (recognized/not recognized)
- Strengths and deficiencies of each execution (free text)
- Overall impression of the element

To evaluate the consistency of these evaluations, inter-rater reliability was calculated using Fleiss’ Kappa. The analysis indicated a Kappa value of 0.44, representing moderate agreement (11).

Separately, the JSS was used to extract three-dimensional skeletal coordinates and automatically detect and classify each trial based on posture and joint angles during the performance. Finally, the JSS outputs were compared with the human judges’ evaluations to examine patterns in recognition and differences in criteria between the JSS and human judges.

## 3 Results

### 3.1 All judges’ results

 Table 2 summarizes the recognition outcomes assigned by the JSS and judges for each trial. The 46 trials were grouped into five outcome categories: (a) recognized by both the JSS and all judges (*n* = 5), (b) not recognized by the JSS and all judges (*n* = 9), (c) recognized by the JSS but not by any of the judges (*n* = 3), (d) recognized by all judges but not by the JSS (*n* = 1), and (e) mixed decisions, with disagreement between the JSS and the judges and/or among the judges themselves (*n* = 28). Among the 28 trials in category (e), the JSS recognized all 7 trials that were recognized by four judges. For the remaining trials, the JSS recognized three out of four trials recognized by three judges, four out of eight trials recognized by two judges, and two out of nine trials recognized by only one judge.

**Table 2:** Recognition outcomes assigned by the JSS and judges.

Trial No.	Judge E	Judge F	Judge G	Judge H	Judge I
(a) Recognized by both the JSS and judges					
2	Arch, head, rear	Arch, head, rear	Arch	Arch	Arch
10	Arch, head, rear	Arch, head, rear	Split	Arch	Rear
17	Arch, head, rear	Arch, head, rear	Split	Arch, rear angle	Arch, rear
18	Arch, head, rear	Arch, head, rear	Split	Arch	Arch
37	Arch, head, rear	Arch, head, rear	Arch	All	Arch, head, rear
(b) Not recognized by both the JSS and judges					
13	All	All	All	All	All
25	All	All	All	All	All
27	Rear	All	Arch, head	All	Arch, head
28	All	All	Arch, head	Arch, head	All
30	Rear	All	Arch, head	Arch, head	Arch, head
31	Rear	All	Arch, head	Arch, head	Arch, head
40	Rear	All	Arch, head	Arch, head	Arch, head
42	Rear	All	Arch, head	Arch, head	All
44	Rear	All	Arch, head	All	All
(c) Recognized only by the JSS.					
19	Rear			Arch, head	
24	Rear	Rear	Arch, head		Arch, head
46	Rear	All	Arch, head	Arch, head	All
(d) Recognized only by the judges					
38	Arch, rear	Arch, rear	Arch, rear	Rear	Arch, rear

Entries indicate the aspects identified by each judge when rendering their recognition decision. For trials recognized by all judges (categories a and d), the listed items represent aspects considered well-executed; for trials not recognized (categories b and c), they represent aspects considered insufficient. “Split,” “Arch,” “Head,” “Rear,” and “Rear angle” correspond to the recognition criteria defined in Section 2.2. “Rear” indicates the relative vertical position of the rear-leg apex to the head, and “Rear angle” indicates the rear-knee flexion angle. “All” indicates that all primary recognition criteria were identified for that trial.

In addition, when rendering their recognition decisions, the judges recorded, for each trial, aspects they considered well-executed and insufficient.

Among trials recognized by both the JSS and the judges (category a), arch was most frequently identified as well-executed (21/25 evaluations), followed by rear (14/25) and head (12/25).

By contrast, for trials not recognized by either the JSS or the judges (category b), arch and head were most frequently identified as insufficient (39/45 evaluations each), followed by rear (29/45).

In trials recognized only by the judges (category d), rear was consistently identified as well-executed (5/5 evaluations).

### 3.2 Trials recognized by both the JSS and judges

Five trials were recognized by both the JSS and judges (Table 3). Their quantitative characteristics were as follows. The average split angle was 186.8 ± 5.9°, with all trials well above the 170° recognition threshold. The average arch angle was 141.8 ± 8.2°, indicating a clear trunk/head extension. The average vertical position of the rear toe was 7.0 ± 4.4 cm above the vertex of the head, and in every trial, the toe exceeded the head. The average rear-knee flexion angle was 119.6 ± 8.1°, demonstrating marked knee flexion. The average height of the rear knee was 1.2 ± 2.9 cm above the horizontal line through the hip joint (hip level), indicating that in most trials the rear leg as a whole was lifted.

**Table 3:** Trials recognized by both the JSS and judges .

Trial No.	Split angle (°)	Arch angle (°)	Rear foot position (cm)	Rear knee angle (°)	Rear knee position (cm)
2	176	137	5	133	2
10	187	131	2	121	−4
17	193	154	14	108	5
18	187	139	4	120	2
37	191	148	10	116	1
Mean ± SD	186.8 ± 5.9	141.8 ± 8.2	7.0 ± 4.4	119.6 ± 8.1	1.2 ± 2.9

Quantitative characteristics were recorded by the JSS.

### 3.3 Trials not recognized by both the JSS and judges

Nine trials were not recognized by both the JSS and judges (Table 4). Their quantitative characteristics were as follows. The average split angle was 169.6 ± 5.5°, and 67% of the trials met the 170° recognition threshold. The average arch angle was 127.3 ± 4.6°, indicating the presence of a backward-arched posture. The average vertical position of the rear toe was −7.2 ± 2.4 cm relative to the vertex of the head; consequently, no trial had the toe above head level. The average rear-knee flexion angle was 118.1 ± 8.2°, indicating knee flexion. The average height of the rear knee was −7.7 ± 1.8 cm relative to hip level, so all trials were below the horizontal line through the hip. Taken together, these findings indicate that in trials not recognized by either the JSS or the judges, multiple component criteria were not achieved.

**Table 4:** Trials not recognized by both the JSS and judges.

Trial No.	Split angle (°)	Arch angle (°)	Rear foot position (cm)	Rear knee angle (°)	Rear knee position (cm)
13	174	126	−6	134	−4
25	164	126	−5	127	−6
27	160	130	−13	106	−9
28	162	127	−8	119	−7
30	172	132	−8	118	−9
31	172	132	−7	108	−10
40	175	124	−8	116	−8
42	172	117	−6	120	−7
44	175	132	−4	115	−9
Mean ± SD	169.6 ± 5.5	127.3 ± 4.6	−7.2 ± 2.4	118.1 ± 8.2	−7.7 ± 1.8

Quantitative characteristics were recorded by the JSS.

### 3.4 Trials recognized only by the JSS

Three trials were recognized by the JSS but not by the judges (Table 5). Their quantitative characteristics were as follows. The average split angle was 174.7 ± 1.2°, with all trials meeting the 170° threshold. The average arch angle was 139.7 ± 1.2°, indicating a clear backward arch. The average vertical position of the rear toe was −2.3 ± 1.2 cm relative to the vertex of the head; thus, in every trial, the toe remained below head level. The average rear-knee flexion angle was 112.3 ± 0.5°, indicating knee flexion. The average height of the rear knee was −6.3 ± 3.8 cm relative to hip level, so all trials were below the horizontal line through the hip.

**Table 5:** Trials recognized only by the JSS .

Trial No.	Split angle (°)	Arch angle (°)	Rear foot position (cm)	Rear knee angle (°)	Rear knee position (cm)
19	175	138	−1	112	−1
24	176	140	−4	112	−9
46	173	141	−2	113	−9
Mean ± SD	174.7 ± 1.2	139.7 ± 1.2	−2.3 ± 1.2	112.3 ± 0.5	−6.3 ± 3.8

Quantitative characteristics were recorded by the JSS.

### 3.5 Trials recognized only by the judges

One trial was recognized by the judges but not by the JSS (Table 6). Its quantitative characteristics were as follows. The split angle was 187°, well above the 170° threshold. The arch angle was 137°, indicating a clear backward arch. The vertical position of the rear toe was −3 cm relative to the vertex of the head, i.e., the toe remained below head level. The rear-knee flexion angle was 127°, indicating marked knee flexion. The height of the rear knee was 1 cm above hip level, indicating that the trailing leg was lifted.

**Table 6:** A trial recognized only by the judges .

Trial No.	Split angle (°)	Arch angle (°)	Rear foot position (cm)	Rear knee angle (°)	Rear knee position (cm)
38	187	137	−3	127	1

Quantitative characteristics were recorded by the JSS.

Taken together, these findings indicate that in this judge-recognized trial, all but one component criterion was achieved, that is, the requirement that the rear toe exceed head level.

### 3.6 Arm position

**Figure 4:**
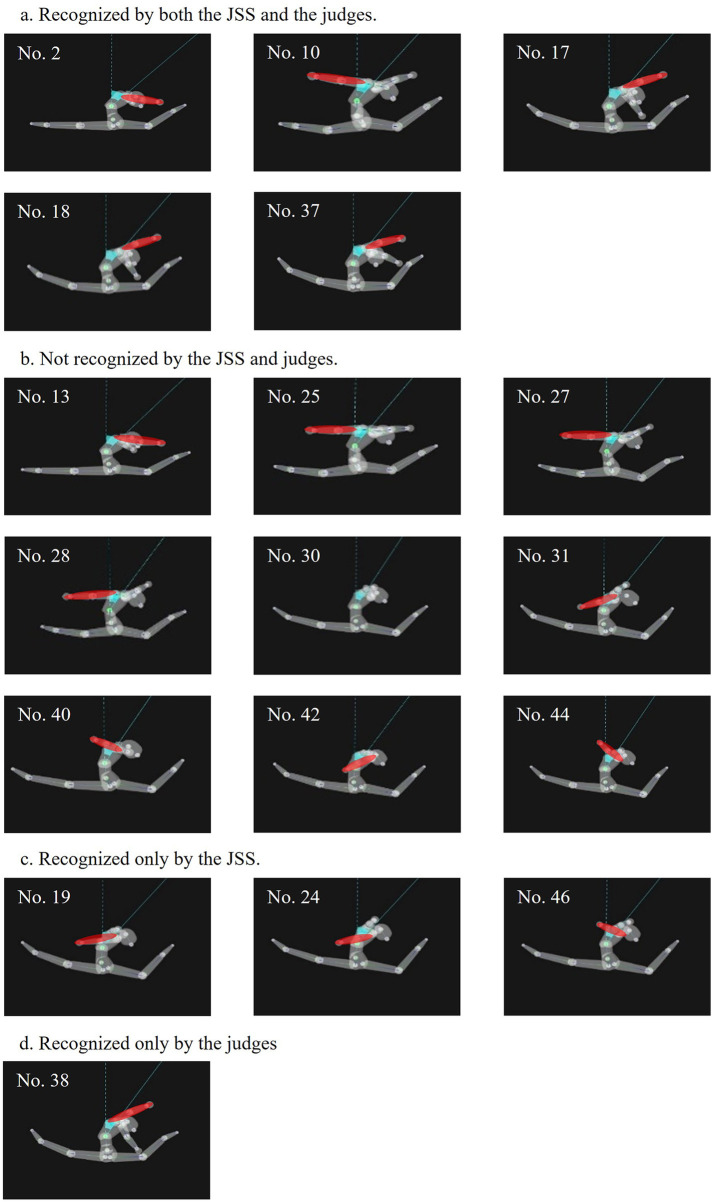
Arm positions during the switch leap to ring position on the balance beam. The 18 trials included in the analysis are presented according to recognition outcome: **(a)** recognized by both the JSS and the judges, **(b)** not recognized by either, **(c)** recognized only by the JSS, and **(d)** recognized only by the judges. The arm segments are highlighted in red.

Figure 4 shows arm positions in the sagittal plane. In trials that were not recognized (e.g., Figures 4b,d), the arms were positioned anterior to the trunk, whereas in recognized trials, they were elevated in line with the head or slightly posterior.

## 4 Discussion

The present findings were generally consistent with our hypotheses: first, that recognition is associated with the integrated configuration of multiple body components, and second, that the JSS tends to yield a higher recognition rate than human judges in borderline cases. These findings suggest three factors that may be associated with recognition of the switch leap to ring position. First, the magnitude of the split angle not only represented as a numeric threshold but also contributed to a visual effect that shapes the overall impression of the element and judges’ subjective evaluations. Second, the coordinated interplay among multiple morphological features aligned with the judges’ idealized whole-body posture, potentially increasing evaluations and the likelihood of recognition.

Third, efforts that enhance visibility, i.e., perceptual legibility of the trunk and trailing leg, may have influenced judges’ perception and recognition decisions regardless of whether individual criteria were strictly met. In what follows, we discuss these three aspects in turn and elucidate the evaluative structure underlying the decision tendencies of both the JSS and judges.

### 4.1 Relationship between split angle and subjective evaluation

In trials recognized by both the JSS and the judges, the mean split angle was 186.8°, exceeding the 170° threshold and contributing to a visually expansive form (Table 3). By contrast, in trials not recognized by either, the mean split angle was 169.6°, and three of the nine trials fell below 170°. Moreover, none of these trials reached the near 180° amplitudes observed in the recognized group (Table 4). These observations suggest that recognition is not determined solely by surpassing the threshold value, but may also be related to achieving a greater amplitude that is perceived as visually more salient.

The present findings may be discussed within the theoretical framework of “processing fluency” (12), which suggests that observers tend to render more positive evaluations to forms that are visually easy to process, such as those exhibiting symmetry and smoothness. In this context, trials with larger split angles may have produced silhouettes that were more perceptually legible, potentially facilitating positive esthetic appraisals by judges. Moreover, in gymnastics more broadly, beyond morphological completeness, visually striking postures strongly shape esthetic appraisal; visual impact sits at the core of impression formation (9). Viewed alongside this literature, the present findings suggest that a larger split angle not only meets the recognition threshold but also facilitates a posture a posture that renders the satisfaction of other criteria, such as the rear-leg apex height and an arched posture, more readily perceived by judges.

Furthermore, Mann (13) pointed out that the visually perceived world and the visual information actually used for motor control might differ, indicating dissociation between the “subjective” visual world and the information guiding action, an observation that is consistent with our results. In other words, trials that judges deemed well-executed did not necessarily satisfy every numerical criterion perfectly; rather, the strong visual impression created by a large split angle may have perceptually compensated for shortfalls in other elements, making the skill appear “ideal.” Accordingly, in evaluating this element, the split angle may serve not only as a checklist item but also as a visual factor shaping overall impression; a higher split angle may have contributed to the perceptual impression that other recognition requirements were satisfied. Therefore, in practice, execution could focus not only on meeting numerical thresholds but also present a visually unambiguous whole-body form that judges are more likely to perceive as “ideal.”

### 4.2 Interrelations among morphological features leading to an ideal posture

In the trials recognized by both the JSS and the judges, the measured morphological variable split angle, arch angle, rear foot position, rear knee angle, and rear knee position—were expressed concurrently. In trials where the rear knee was maintained above hip level, characteristic numerical values were also observed for the arch angle and rear foot position. The mean arch angle in the recognized trials was 141.8 ± 8.2°, whereas it was 127.3 ± 4.6° in the non-recognized trials. Similarly, the mean rear foot position was 7.0 ± 4.4 cm in the recognized trials, indicating that the rear foot was positioned above hip level, whereas it was −7.2 ± 2.4 cm in the non-recognized trials (Tables 3, 4).

Given that no inferential statistical testing was performed and the sample size was limited, these differences should be interpreted descriptively. Nevertheless, the recognized trials appeared to share a coordinated postural configuration in which the split angle, arch angle, and rear-leg elevation were realized simultaneously, suggesting that successful recognition may depend on the coordinated organization of multiple body segments under task constraints, rather than on a single isolated criterion (16).

In these trials, judges collectively evaluated the arch/head, rear apex, and split as well-executed (Table 2). This pattern suggests that judges’ decisions may not rely solely on the achievement of a single local shape, but may relate to the clarity and unity of the whole-body posture formed through the concurrent expression of multiple morphological features.

Furthermore, in forming the arch, not only trunk extension but also upper-limb positioning may influence visual impression (Figure 4). Although upper-limb position was not quantitatively analyzed in the present study, qualitative observation of the video recordings indicated that, in four of the five recognized trials, the arms tended to be positioned posterior to the head. Such arm positioning may have visually accentuated the contour of the arch in conjunction with neck alignment. Posterior arm elevation may also be related to trunk extension; however, this relationship was not quantitatively examined in the present study and warrants further kinematic investigation.

Previous literature has suggested that contextual factors and athlete characteristics may influence judging decisions. While these aspects were not directly examined in the present dataset, they are considered relevant as a general theoretical perspective.

Taken together, recognition of this element may not be secured by satisfying a single numerical criterion alone but may be associated with the simultaneous realization of multiple morphological features that contribute to a clear and unified whole-body posture. The rear-knee angle and position, the arch, and arm elevation may not operate independently, but rather appear as components of a consistent postural configuration that shapes visual impression.

### 4.3 Impression formation and “visibility”

Recognition decisions may depend not only on whether each requirement meets its threshold value, but also on how clearly the overall posture is perceived as a ring shape. Supporting our hypothesis, the JSS resulted in a higher recognition rate than human judges in these borderline trials, with three trials recognized only by the JSS (Table 5) compared to only one trial recognized only by the judges (Table 6). In particular, when comparing the trials recognized only by the JSS (Table 5) with the trial recognized only by the judges (Table 6), the arch angle was 139.7 ± 1.2° in the JSS-only trials and 137° in the judge-only trial. Similarly, the rear foot position was −2.3 ± 1.2 cm in the JSS-only trials and −3 cm in the judge-only trial. Although these values were numerically comparable, the recognition outcomes differed. This observation suggests that even when individual angles or positional values approach the required criteria, a trial may be less likely to be recognized if the posture is not clearly perceived as a ring shape. Furthermore, as observed in the trials recognized by either the JSS (Table 5) or the judges (Table 6) alone, recognition was granted in some cases even when the rear foot was slightly below head level. This suggests that each recognition system may incorporate a degree of flexibility, evaluating the overall posture rather than relying solely on individual numerical criteria.

By contrast, the rear knee position was +1 cm in the judge-only recognized trial, whereas it was −6.3 ± 3.8 cm in the JSS-only trials. This difference may reflect whether the trailing leg was sufficiently elevated above the hip level, a feature related to the apparent completeness of the posture. A higher trailing leg may contribute to the visual clarity of the ring position, potentially making the required configuration easier to discern. This finding may indicate that the visual clarity of the ring posture was involved in the recognition decision.

Furthermore, in the trials not recognized by the judges, the upper limbs were frequently positioned forward (Figure 4). Although these observations are qualitative rather than based on quantitative measurements and arm position is not a codified requirement, its placement could potentially influence the visual continuity of the arch line. This contrast might reflect a difference in perceptual processing: while the JSS tracks skeletal coordinates as discrete “points,” human judges may take more into account the overall visual configuration of the movement. Therefore, even if arm positions do not affect the JSS's coordinate-based logic, they could potentially influence human perception by affecting the structural coherence of the ring shape. Conversely, a single, visually salient feature—such as an exceptional split angle— might contribute to a perceptual impression where minor deficits in other criteria become less apparent, leading to the perceiving the overall form as a unified “gestalt.”

Taken together, whereas the JSS evaluates performance based on localized kinematic parameters, judges may place greater emphasis on whether the posture appears as a coherent ring configuration when viewed as a whole. Because the JSS computes the arch angle directly as a numerical value, its evaluation is less likely to be affected by peripheral features such as arm position. This contrast may in part reflect a difference in emphasis, with the JSS relying on discrete numerical criteria and human judges potentially attending more to the overall visual configuration.

Accordingly, increasing the likelihood of recognition of the switch leap to ring may require not only meeting numerical thresholds for angles and heights, but also presenting these elements in a manner that makes the overall structure visually clear. Adjusting auxiliary features such as arm placement and trunk extension may serve as practical means of enhancing the visual clarity of the posture.

Such a decision process may be interpreted in light of processing fluency theory (12), which proposes that stimuli that are easier to process perceptually tend to be evaluated more favorably. In the present context, trials in which the required elements were integrated into a clearly organized configuration may have been more readily perceived as constituting a valid ring position.

### 4.4 Limitations

Several limitations should be considered when interpreting the findings of this study. First, the sample size was small, consisting of 46 borderline trials from four gymnasts. Since this dataset includes multiple trials per athlete, the observations lack strict statistical independence. While this study intentionally selected trials where judgments were most likely to be divided among human judges to represent a “gray zone,” individual-specific execution patterns may have influenced the kinematic results. Therefore, these findings should be interpreted with caution regarding their generalizability.

Second, regarding the video sampling rate, the JSS utilizes 30 Hz as its operational standard, which is the frequency required for automated skill recognition in major international competitions, including the Olympic Games. While this rate is robust for practical scoring applications, it is important to acknowledge that, from a strictly kinematic perspective, it may not capture the absolute maximum point of extension achieved between frames. Although human judges are subject to similar or even greater perceptual limitations, this technical specification should be noted when interpreting precise kinematic thresholds.

Finally, a clear distinction must be made between empirical evidence and interpretative hypotheses. Our findings regarding variables with official technical requirements, such as the split and arch angles, are grounded in quantitative kinematic data. In contrast, observations of auxiliary features, such as arm position, remain qualitative. The potential influence of these features on visual impression and judging decisions should be treated as a hypothesis requiring further empirical verification.

## 5 Conclusions

This study focused on the switch leap to ring position—an element within the dance group of women's artistic gymnastics that frequently results in divergent recognition outcomes. Using the AI-based JSS, this study attempted to identify kinematic characteristics associated with recognition and discuss postural factors that may contribute to consistent evaluation.

In trials recognized by both the JSS and judges, three elements were consistently achieved at high levels: split, arch and head, and rear (apex of the trailing leg). In addition, the trials recognized by the judges exhibited a tendency for the rear-knee position to be maintained above hip level. Furthermore, within the arch/head configuration, arm placement appeared to facilitate recognition by modulating the visibility and overall impression of the posture.

These findings suggest that for coaching and skill execution, meeting individual numeric thresholds alone may not be the sole determinant of recognition. Enhancing the coordination and visibility of key morphological features to create a visually coherent whole-body posture could be a beneficial factor for improving the likelihood that the switch leap to ring position is recognized.

## Data Availability

The raw data supporting the conclusions of this article will be made available by the authors, without undue reservation.
